# Characterizing coral skeleton mineralogy with Raman spectroscopy

**DOI:** 10.1038/s41467-018-07601-3

**Published:** 2018-12-14

**Authors:** Thomas M. DeCarlo

**Affiliations:** 1 School of Earth Sciences and Oceans Graduate School, The University of Western Australia, 35 Stirling Highway, Crawley, WA 6009 Australia; 2 Australian Research Council Centre of Excellence for Coral Reef Studies, The University of Western Australia, Crawley, WA 6009 Australia

**ARISING FROM** A. Akiva et al. *Nature Communications* 10.1038/s41467-018-04285-7 (2018)

Knowledge of the mineralogy of coral skeletons is essential for understanding the sensitivity of corals to climate change, particularly ocean acidification. Although it is well known that scleractinian coral skeletons are composed of the calcium carbonate mineral aragonite, some authors have challenged the classical notion that the aragonite skeleton is precipitated directly from seawater, and instead suggest that other pre-cursor phases exist prior to transformation into aragonite. In a recent *Nature Communications* article, Akiva et al.^1^ purport to show that both coral planulae (free-swimming coral larvae) and polyps of juvenile corals precipitate amorphous calcium carbonate (ACC) particles that are then transformed into the aragonite crystals that form the skeleton. A key piece of evidence in their paper for the presence of ACC was Raman spectroscopy characterization of newly formed skeletons. However, their Raman data do not actually show ACC, but rather indicate the presence of calcite, a crystalline calcium carbonate. Whether corals build their skeletons from ACC particles remains an interesting hypothesis, yet it is far from being supported by conclusive evidence.

Raman spectroscopy is a geochemical tool capable of accurately and rapidly identifying sample mineralogy^2^. This technique is particularly effective at distinguishing the various calcium carbonate minerals including calcite, aragonite, and vaterite, and amorphous phases^3–9^. The Raman spectra of all calcium carbonates have a strong ν_1_ peak between 1080–1090 cm^−1^ that represents the symmetric stretching of C–O bonds^8,10^ (Fig. 1a). Other C–O bond peaks, such as in-plane bend (ν_4_) in the 700–720 cm^−1^ region (Fig. 1a), are effective for discriminating between calcite and aragonite^4,8,11^. The ν_1_ and ν_4_ peaks are called “internal modes” because they originate from vibrations between the C and O of carbonate (CO_3_). In addition, the Raman spectra of crystalline calcium carbonates contain “lattice modes” (or “external modes”) in the <400 cm^−1^ region that result from vibrations between molecules in the lattice (Fig. 1b), and these lattice modes are also mineral-specific^8,10^.

**Fig. 1:**
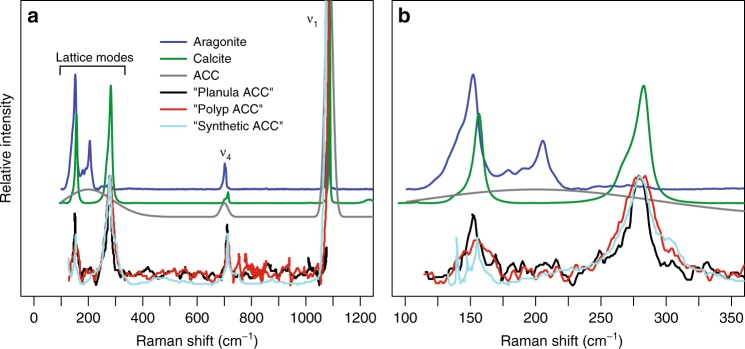
Reference Raman spectra of calcium carbonates: aragonite (blue), calcite (green), and ACC (gray). **a** shows the broad spectrum from 0 to 1300 cm^−1^ that includes both lattice and internal modes, and **b** shows the narrower 100–400 cm^−1^ lattice-mode region. Internal modes ν_1_ and ν_4_ are labelled, and the region of lattice modes for aragonite and calcite is indicated. The “Planula ACC” (black), “Polyp ACC” (red), and “Synthetic ACC” (cyan) lines show the data from Akiva et al.^1^ interpreted as spectra of ACC. These three spectra all have distinct lattice mode peaks, clearly demonstrating they are not ACC, but rather a crystalline calcium carbonate consistent with calcite

ACC is best identified in Raman spectroscopy based on the width of the ν_1_ peak^7,12^. This is because ACC is highly disordered, meaning that the carbonate molecules are not arranged in a particular pattern^13^. In this disordered phase, the bonding environment around the carbonates is highly variable, and this causes deviations in the lengths of C–O bonds. Differences in C–O bond lengths cause variations in their vibrational frequency, thus affecting the ν_1_ position in the Raman spectrum. Therefore, the broad distribution of C–O bond lengths in ACC causes the ν_1_ peak to be much wider than most crystalline calcium carbonates^7^. High-Mg calcite may also have a broad ν_1_ peak due to the disorder caused by Mg substituting for Ca, thus requiring knowledge of the Mg content to distinguish between high-Mg calcite and ACC using ν_1_ peak width^12^. Conversely, the lattice modes in the <400 cm^−1^ region of the Raman spectrum cannot be used to identify ACC. This is because ACC–by definition–does not have a crystal lattice and therefore does not have distinct peaks in this part of the spectrum^7,12–14^. While a truly amorphous phase would be expected to have a flat spectrum in the lattice mode region, some ordering of carbonate ions in ACC can cause a broad increase in the background intensity, but crucially, still without distinct lattice peaks^13^. However, any “hump” in the <400 cm^−1^ region due to slight carbonate ordering must not be confused with a broad increase in the background intensity due to fluorescence, especially when conducting measurements in seawater or through biological tissues.

The high sensitivity of Raman spectroscopy to distinguish between crystalline and amorphous phases at high-resolution and in vivo makes it the most powerful tool used in the Akiva et al. study^1^ to identify ACC. Their reported finding of ACC in the early life stages of corals with Raman spectroscopy therefore has important ramifications for how the scientific community understands coral growth, and for the interpretation of a variety of geochemical climate proxies applied to coral skeletons. However, Akiva et al.^1^ used the presence of lattice modes in the <400 cm^−1^ region to identify a purported ACC phase. As described above, because the material that they analyzed has distinct lattice modes, it cannot be ACC (Fig. 1). In fact, their Raman spectra clearly reveal that all the materials they studied are crystalline. While they apparently used a synthetic material as a standard for identifying ACC in Raman spectra of coral skeletons, no evidence was provided to verify that the synthetic material was ACC, and their own Raman data are proof that the synthetic material was a crystalline calcium carbonate.

The “ACC” samples in the Akiva et al.^1^ study all show peaks around Raman shifts of 150 and 280 cm^−1^, the latter being clearly different from aragonite. These lattice-mode peaks are entirely consistent with the presence of calcite (Fig. 1). While two previous studies used Raman spectroscopy to probe juvenile coral skeletons for calcite and found none^15,16^, another study using Raman spectroscopy identified both calcite and aragonite in the very earliest stages of polyp settlement^17^. Since calcite is more stable than aragonite^18^, it is unlikely that calcite could be a pre-cursor to the aragonite found in coral skeletons. An alternative explanation is that the calcite has enough magnesium to increase its solubility (decreased stability) to the extent that it could–in theory–be a pre-cursor to aragonite^18^. This explanation is consistent with the high levels of magnesium that Akiva et al.^1^ reported in the earliest formed skeletons, and with two other studies reporting high Mg concentrations in the basal plates of newly settled polyps^17,19^. Yet, it is difficult to reconcile a calcite pre-cursor to skeletal aragonite because calcite and aragonite incorporate trace elements at very different concentrations, and the geochemistry of coral skeletons–including juveniles–is consistent with direct precipitation of aragonite from seawater^11,20–22^. As described previously^17,19^, this high-Mg phase in the initial basal plate may be an important step in coral settlement, but it is likely restricted to a small amount of the initial skeleton and is not representative of the process by which most of the skeleton is formed.

Identifying the mechanisms of skeletal formation in the earliest life stages of corals is critical to advancing our knowledge of how corals will respond to ocean acidification. Akiva et al.^1^ present interesting results showing different mineral phases besides aragonite in both coral planulae and juvenile polyps. However, contrary to the interpretation in the original paper^1^, their Raman data clearly demonstrate that this non-aragonite material is not ACC, but rather it is crystalline, likely a high-Mg calcite. It should be noted that Akiva et al.^1^ also used NMR to characterize their “ACC”, and they are not the first to claim to find ACC in coral skeletons based on a combination of Raman and NMR data^23^. Yet, as described by DeCarlo et al.^24^, the Raman data in this previous study by Von Euw et al.^23^ were also clearly not ACC (based on ν_1_ peak width). Thus, uncertainties remain in how to reconcile Raman and NMR characterizations of mineral phases in coral skeletons. The results of Akiva et al.^1^ are potentially important findings for understanding coral biomineralization, but additional analyses are certainly needed to more definitively identify non-aragonite calcium carbonates in the earliest stages of coral growth.

## Methods

Reference aragonite and calcite spectra were acquired from the RRUFF online database (ID R080142 and R150075, respectively) and plotted in Fig. 1. An ACC spectrum (gray line in Fig. 1) was illustrated based on descriptions in the literature^7,13,14^ of wide internal modes (e.g. ν_1_ full width at half maximum intensity >20 cm^−1^)^7^, and an absence of distinct lattice mode peaks but with a broad background at <400 cm^−1^ due to some ordering of carbonate^13^. The corresponding authors of Akiva et al.^1^ did not respond to requests for the raw Raman data, so their “ACC” spectra were extracted with the software Datathief (http://www.datathief.org). This was conducted on their Fig. 4 and their Supplementary Fig. 3 separately, and plotted here in Fig. 1a and Fig. 1b, respectively. In Fig. 1a, the Akiva et al.^1^ data are only plotted below approximately 1080 cm^−1^ because it was not possible to extract the full ν_1_ peak from their Fig. 4 since the spectra were overlapping.

## Data Availability

Raman spectra of aragonite and calcite are available at http://rruff.info/Aragonite/R080142 and http://rruff.info/Calcite/R150075.
